# A risk stratification model for predicting brain metastasis and brain screening benefit in patients with metastatic triple‐negative breast cancer

**DOI:** 10.1002/cam4.3449

**Published:** 2020-09-18

**Authors:** Mingxi Lin, Yizi Jin, Jia Jin, Biyun Wang, Xichun Hu, Jian Zhang

**Affiliations:** ^1^ Department of Medical Oncology Fudan University Shanghai Cancer Center Shanghai China; ^2^ Department of Oncology Shanghai Medical College Fudan University Shanghai China

**Keywords:** Brain metastasis, Metastatic triple‐negative breast cancer, Nomogram, Overall survival, Prognosis

## Abstract

**Background:**

Patients with metastatic triple‐negative breast cancer (mTNBC) frequently experience brain metastasis. This study aimed to identify prognostic factors and construct a nomogram for predicting brain metastasis possibility and brain screening benefit in mTNBC patients.

**Methods:**

Patients with mTNBC treated at our institution between January 2011 and December 2018 were retrospectively analyzed. Fine and Gray's competing risks model was used to identify independent prognostic factors. By integrating these prognostic factors, a competing risk nomogram and risk stratification model were developed and evaluated with concordance index (C‐index) and calibration curves.

**Results:**

A total of 472 patients were retrospectively analyzed, including 305 patients in the training set, 78 patients in the validation set I and 89 patients in the validation set II. Four clinicopathological factors were identified as independent prognostic factors in the nomogram: lung metastasis, number of metastatic organ sites, hilar/mediastinal lymph node metastasis and KI‐67 index. The C‐indexes and calibration plots showed that the nomogram exhibited a sufficient level of discrimination. A risk stratification was further generated to divide all the patients into three prognostic groups. The cumulative incidence of brain metastasis at 18 months was 5.3% (95% confidence interval [CI], 2.5%‐9.7%) for patients in the low‐risk group, while 14.3% (95% CI, 9.3%‐20.4%) for patients with intermediate risk and 34.3% (95% CI, 26.8%‐41.9%) for patients with high risk. Routine brain MRI screening improved overall survival in high‐risk group (HR 0.67, 95% CI 0.46‐0.98, *P* = .039), but not in low‐risk group (HR 0.93, 95% CI 0.57‐1.49, *P* = .751) and intermediate‐risk group (HR 0.83, 95% CI 0.55‐1.27, *P* = .386).

**Conclusions:**

We have developed a robust tool that is able to predict subsequent brain metastasis in mTNBC patients. Our model will allow selection of patients at high risk for brain metastasis who might benefit from routine bran MRI screening.

## Graphical abstract

This study found four risk factors for the development of brain metastasis in mTNBC patients: lung metastasis, number of metastatic organ sites, hilar/mediastinal lymph node metastasis and KI‐67 index. By integrating these prognostic factors, a competing risk nomogram was generated to predict individualized brain metastasis possibility. Our model will allow selection of patients at high risk for brain metastasis who might benefit from routine bran MRI screening.

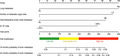

## 1 INTRODUCTION

Among patients diagnosed with breast cancer, 5%‐10% had primary metastasis, and 20%‐30% would develop into metastatic disease.^1^ Metastatic breast cancer (MBC) remains an incurable disease with an estimated 5‐year survival of only 25%.^1^ Approximately, 25% of patients with MBC developed brain metastasis.^2^ The blood‐brain barrier prevents the penetration of anticancer drugs, which may result in reduced drug delivery to the site of brain metastases and insufficient control of the cerebral tumor.^3^ Patients with brain metastasis may suffer from cognitive and sensory dysfunctions, and the mortality within one year of diagnosis is approximately 80%.^4^

Breast cancer has different molecular subtypes and the predilection for brain metastasis varies among different subtypes.^5^ For hormone receptor‐positive MBC, only 14% of patients developed brain metastasis, with a median survival time of 9‐10 months afterward.^6^, ^7^ For patients with human epidermal growth factor receptor‐2 (HER2)‐positive MBC, about 34% developed brain metastasis, with an estimated median survival of 11 months afterward.^6^ Despite the high occurrence of brain metastasis among patients with HER2‐positive MBC, the addition of small molecule anti‐HER2 agents to trastuzumab results in increased overall survival for this population.^8^ However, the outcome is different for patients with metastatic triple‐negative breast cancer (mTNBC). Lin et al reported that 46% of mTNBC patients in their study developed brain metastasis, with a median survival of only 4.9 months.^9^ Therefore, it is of great need to identify a novel regime for the treatment of brain metastasis in mTNBC patients. Nowadays, local treatment, such as stereotactic radiosurgery (SRS), is the first option for the management of brain metastasis.^10^, ^11^ As local treatments are limited to patients with less extensive central nervous system disease, early detection of brain metastasis might improve patients’ outcome. Therefore, risk stratification tool for predicting subsequent brain metastasis is needed. Brain screening of patients with high risk for brain metastasis might lead to early detection and better outcome.

In recent years, the nomogram has become a commonly used predictive tool. The nomogram generates individualized risk prediction by combining risk factors. Nomograms were developed in a few previous studies to predict subsequent brain metastasis probability in patients with MBC,^12^, ^13^ but none of these studies focused on mTNBC or accounted appropriately for competing risks. The competing risks situation arose when an individual can experience more than one type of event and the occurrence of one type of event hindered the occurrence of other type of event.^14^ For example, the occurrence of death hindered the occurrence of brain metastasis. In the presence of competing risks, conventional Kaplan‐Meier and Cox methods may be inappropriate.

The objective of this study is to develop and validate a competing risk nomogram for predicting subsequent brain metastasis in mTNBC patients and stratify the high‐risk group that might benefit from brain MRI screening.

## 2 MATERIALS AND METHODS

### 2.1 Data sources and study population

A retrospective study was conducted on a primary cohort of mTNBC patients who received first‐line treatment at Fudan University Shanghai Cancer Center (FUSCC) from Jan 1, 2011, to Dec 31, 2018. All of the patients had histologically confirmed TNBC with radiographic and/or histological evidence of advanced disease. Estrogen receptor (ER)‐negative status and progesterone receptor (PgR)‐negative status were defined as immunohistochemistry (IHC) results showing < 1% expression. HER2‐negative status was defined as a score of 0 or 1 based on the IHC results or negative results in fluorescence in situ hybridization (FISH). For patients who underwent biopsies at the metastatic sites, we redefined the molecular subtypes (including ER, PgR, HER‐2, and KI‐67) based on the metastatic lesions. Patients with brain metastasis at the first metastasis diagnosis, patients with insufficient (<3 months) follow‐up times and patients with history of other malignancies were excluded. The included patients formed the training cohort of this study.

From Jan 1, 2015 to Dec 31, 2018, two independent cohort of mTNBC patients who received first‐line treatment in prospective clinical trials NCT02341911 and NCT02546934 at FUSCC were retrospectively studied, using the same inclusion and exclusion criteria. These patients formed the validation set I and validation set II of this study, respectively.

### 2.2 Study variables

To develop a practical nomogram, we considered 18 routinely available clinicopathological covariates proven to predict brain metastasis for patients with breast cancer in prior studies.^15^, ^16^, ^17^, ^18^ All relevant information was retrieved retrospectively from the FUSCC electronic database and consisted of (a) demographic data including age at first metastasis diagnosis and menopausal status; (b) pathologic data including histological subtype, histological grade, KI‐67 index, and stage at initial diagnosis of cancer; (c) treatment regimen data including neoadjuvant chemotherapy, initial surgery, prior neo/adjuvant chemotherapy, adjuvant radiotherapy, receiving baseline brain MRI screening or not; (d) laboratory data including lactate dehydrogenase (LDH), cancer antigen 125 (CA125), cancer antigen 153 (CA153), and carcinoembryonic antigen (CEA); (5) metastasis characteristics including initial site of metastasis, presence of visceral metastasis, number of metastatic organ sites, and time between breast surgery and metastatic disease.

The initial site of metastasis was established by biopsy and/or the clinical radiological report. The clinical radiological report included computerized tomography (CT) for the chest, abdomen, pelvis, and bone, and brain magnetic resonance imaging (MRI) with gadolinium contrast enhancement. Lymph nodes were considered metastatic if they were enlarged (short axis ≥ 10 mm) with abnormal morphology.

Laboratory covariates were dichotomized based on the clinical cut‐off values (categorization as below or above the upper limit of normal at our institution) and had to be measured within the 2 weeks preceding the first‐line treatment of metastatic disease. Other continuous covariates were converted into categorical covariates based on the acknowledged cut‐off number (for age) or median value (for the KI‐67 index). Two researchers collected the data independently and disagreements were resolved through discussions with a third expert.

### 2.3 Primary outcome

The primary outcome was brain metastasis‐free survival, which was defined as the time elapsed from the date of first metastasis diagnosis to the date of brain metastasis diagnosis. Brain metastasis was defined as parenchymal metastasis (leptomeningeal metastasis was not included) and death before brain metastasis was considered a competing event. Patients without brain metastasis or death events were censored at the last follow‐up. Diagnostic workup for brain metastasis was initiated in symptomatic patients using brain MRI screening. Typical indications for imaging were unexplained headache, sensory or motoric peripheral or central neurological symptoms. Brain MRI screening is not mandatory in asymptomatic patients. However, patients were recommended to take routine brain MRI screening every 3‐4 months. The date of the last follow‐up was 31 December 2019.

### 2.4 Statistical analysis

Descriptive statistics were applied to summarize patients’ baseline demographic and clinical characteristics of the three cohorts. In consideration of potential competitive risk (death before brain metastasis), the Fine and Gray's competing risks proportional hazards regression model was applied to assess the independent predictive variables for subsequent brain metastasis.^19^ To visualize the prediction model, a nomogram was constructed using the methods described by Zhang et al^20^ The model's predictive performance was evaluated based on the discrimination performance and the calibration plot. The discrimination for the nomogram is its ability to distinguish a patient with subsequent brain metastasis from a patient without brain metastasis. The discrimination performance was evaluated by estimating Wolber's C‐index (especially used for competing risks models).^21^ A calibration plot graphically compares the model‐predicted probability to the observed incidence.^22^ Bootstrapping taking 2000 resamples was used to calculate the C‐index and generate calibration plots.

A risk stratification was performed on the basis of each patient's total scores in the nomogram. All the patients were divided into three stratums. The cumulative incidence of brain metastasis in different groups was estimated through the cumulative incidence function (CIF), taking into consideration the competing risk of death before brain metastasis. Gray's test was used to compare the differences between groups.^23^ The median overall survival (OS) and the 95% confidence intervals (CIs) were calculated using the Kaplan‐Meier method and compared using the log‐rank test among different groups. Hazard ratios (HRs) with two‐sided 95% confidence intervals were calculated by Cox proportional hazards models to measure the difference in the survival distribution.

All statistical analyses were performed using the Statistical Package for the Social Sciences (SPSS) version 26 and R software version 3.4.2 with the R packages rms, cmprsk, smcfcs, and mstate. All statistical tests were two‐tailed and *P* < .05 was considered statistically significant.

## 3 RESULTS

### 3.1 Clinicopathologic characteristics of patients

A total of 472 patients were retrospectively analyzed, including 305 patients in the training set, 78 patients in the validation set I and 89 patients in the validation set II. The baseline characteristics of the study population are presented in Table 1.

**Table 1:** Patients’ baseline demographic and clinical characteristics

Characteristics	Training set (N = 305)	Validation set I (N = 78)	Validation set II (N = 89)
	No. of patients (%)	No. of patients (%)	No. of patients (%)
Age at metastasis diagnosis, years			
≤40	64 (21.0)	11 (14.1)	16 (18.0)
>40	241 (79.0)	67 (85.9)	73 (82.0)
Menopausal status			
Pre‐ or perimenopause	193 (63.3)	38 (48.7)	31 (34.8)
Postmenopause	112 (36.7)	40 (51.3)	58 (65.2)
Histological subtype			
Invasive ductal	301 (98.7)	74 (94.9)	84 (94.4)
Lobular	3 (1.0)	3 (3.8)	0 (0.0)
Metaplastic	0 (0.0)	1 (1.3)	4 (4.5)
Medullary	1 (0.3)	0 (0.0)	1 (1.1)
Histological grade			
I‐II	84 (27.5)	24 (30.8)	24 (27.0)
III	221 (72.5)	54 (69.2)	65 (73.0)
KI‐67 index			
≤50%	128 (42.0)	48 (61.5)	45 (50.6)
>50%	177 (58.0)	30 (38.5)	44 (49.4)
Stage at initial diagnosis of cancer^a^			
I	33 (10.8)	13 (16.7)	19 (21.3)
II	125 (41.0)	34 (43.6)	45 (50.6)
III	104 (34.1)	25 (32.0)	20 (22.5)
IV	43 (14.1)	6 (7.7)	5 (5.6)
Neoadjuvant chemotherapy^b^			
No	221 (84.4)	63 (87.5)	77 (91.7)
Yes	41 (15.6)	9 (12.5)	7 (8.3)
Initial surgery			
Complete mastectomy	238 (78.1)	60 (76.9)	74 (83.1)
Breast‐conserving surgery	30 (9.8)	12 (15.4)	8 (9.0)
No initial breast surgery	37 (12.1)	6 (7.7)	7 (7.9)
Prior neo/adjuvant chemotherapy^b^			
Anthracycline‐based regimen	32 (12.2)	7 (9.7)	6 (7.1)
Taxanes‐based regimen	8 (3.1)	4 (5.6)	9 (10.7)
Anthracycline & Taxanes‐based regimen	184 (70.2)	56 (77.8)	64 (76.2)
Others	38 (14.5)	5 (6.9)	5 (6.0)
Adjuvant radiotherapy^b^			
No	128 (48.9)	28 (38.9)	48 (57.1)
Yes	134 (51.1)	44 (61.1)	36 (42.9)
Time between breast surgery and metastatic disease			
≤1 year	72 (23.6)	8 (10.3)	13 (14.6)
>1 year	190 (62.3)	64 (82.0)	71 (79.8)
Primary metastatic	43 (14.1)	6 (7.7)	5 (5.6)
Baseline brain MRI screening			
No	128 (42.0)	0 (0.0)	0 (0.0)
Yes	177 (58.0)	78 (100.0)	89 (100.0)
Initial site of mTNBC			
Lung involvement	152 (49.8)	38 (48.7)	50 (56.2)
Liver involvement	78 (25.6)	17 (21.8)	19 (21.3)
Bone involvement	105 (34.2)	28 (35.9)	26 (29.2)
Pleural effusion involvement	45 (14.8)	9 (11.5)	21 (23.6)
Non‐regional LN involvement	172 (56.4)	45 (57.7)	54 (60.7)
Hilar/ mediastinal LN involvement	93 (30.5)	31 (39.7)	36 (40.4)
Visceral metastasis			
No	95 (31.1)	27 (34.6)	26 (29.2)
Yes	210 (68.9)	51 (65.4)	63 (70.8)
Number of metastatic organ sites			
1	138 (45.2)	36 (46.2)	41 (46.1)
≥2	167 (54.8)	42 (53.8)	48 (53.9)
LDH^c^, U/L			
≤250	206 (67.5)	58 (74.4)	67 (75.3)
>250	99 (32.5)	20 (25.6)	22 (24.7)
CA125^c^, U/mL			
≤35	193 (63.3)	53 (67.9)	49 (55.1)
>35	112 (36.7)	25 (32.1)	40 (44.9)
CA153^c^, U/mL			
≤25	207 (67.9)	48 (61.5)	64 (71.9)
>25	98 (32.1)	30 (38.5)	25 (28.1)
CEA^c^, ng/mL			
≤5.20	268 (87.9)	68 (87.2)	73 (82.0)
>5.20	37 (12.1)	10 (12.8)	16 (18.0)

Abbreviations: BM, Brain metastasis; CA125, Cancer antigen 125; CA153, Cancer antigen 153; CEA, Carcinoembryonic antigenLDH, Lactate dehydrogenase; LN, Lymph node; mTNBC, Metastatic triple negative breast cancer.

^a^ Based on the American Joint Committee on Cancer Staging Manual, 8th ed.

^b^ Patients with primary metastatic disease were not included.

^c^ All variables were measured within 2 weeks preceding the first‐line treatment of metastatic disease.

Among the 472 patients, 381 (80.7%) patients were older than 40 years old, while 210 (44.5%) patients were postmenopause. The most common histological subtypes were invasive ductal carcinoma (97.2%) and histological grade III disease (72.0%). Most patients had a heavy burden of metastatic disease at the first metastasis diagnosis. More than half of the patients (54.4%) had two or more metastatic organ sites while 324 (68.6%) patients presented with visceral metastasis. The median follow‐up time was 16.3 (Interquartile range [IQR] 10.1‐25.5) months from metastatic disease diagnosis. About 117 (24.8%) patients developed brain metastasis, whereas 225 (47.7%) patients died before brain metastasis. The remaining 130 (27.5%) patients had no brain metastasis or death at the time of the last follow‐up.

### 3.2 Independent prognostic factors

The univariable analysis results are listed in Table 2. Significant covariates (*P*‐value < 0.05 in the univariable analysis) were included in the multivariable analysis using the Fine and Gray competing risks model. KI‐67 index (>50%: SHR 1.71, 95% CI 1.04‐2.80; ≤50% as a reference), number of metastatic organ sites (≥2 sites: SHR 2.28, 95% CI 1.22‐4.27; 1 site as a reference), lung metastasis (metastasis: SHR 2.66, 95% CI 1.24‐5.70; no metastasis as a reference), and hilar/mediastinal lymph node metastasis (metastasis: SHR 1.70, 95% CI 1.02‐2.84; no metastasis as reference) remained as independent prognostic factors for brain metastasis in the competing risks model (Table 2) and were used to construct a nomogram.

**Table 2:** Univariable and multivariable competing‐risk regression analysis for brain metastasis‐free survival

Characteristics	Univariable Analysis			Multivariable Analysis		
	SHR	95% CI	*P*	SHR	95% CI	*P*
Age at metastasis diagnosis, years						
≤40	1					
>40	0.90	(0.51‐1.57)	.710			
Menopausal status						
Pre‐ or perimenopause	1					
Postmenopause	1.08	(0.67‐1.73)	.760			
Histological subtype						
Invasive ductal	1					
Lobular/metaplastic/medullary	0.90	(0.11‐7.39)	.920			
Histological grade						
I‐II	1					
III	1.07	(0.64‐1.79)	.800			
KI‐67 index						
≤50%	1			1		
>50%	1.65	(1.01‐2.70)	**.047**	1.71	(1.04‐2.80)	**0.035**
Stage at initial diagnosis of cancer^a^						
I	1					
II	0.73	(0.36‐1.48)	.380			
III	0.52	(0.24‐1.12)	.093			
IV	0.94	(0.42‐2.10)	.880			
Neoadjuvant chemotherapy^b^						
No	1					
Yes	1.18	(0.59‐2.44)	.620			
Initial surgery						
Complete mastectomy	1					
Breast‐conserving surgery	1.60	(0.80‐3.22)	.180			
No initial breast surgery	1.71	(0.89‐3.21)	.110			
Prior neo/adjuvant chemotherapy^b^						
Anthracycline‐based regimen	1					
Taxanes‐based regimen	1.08	(0.22‐5.36)	.930			
Anthracycline & Taxanes‐based regimen	0.54	(0.28‐1.04)	.063			
Others	0.81	(0.36‐1.82)	.620			
Adjuvant radiotherapy^b^						
No	1					
Yes	0.83	(0.50‐1.39)	.480			
Time between breast surgery and metastatic disease						
≤1 year	1					
>1 year	1.54	(0.82‐2.90)	.180			
Primary metastatic	1.93	(0.89‐4.17)	.094			
Baseline brain MRI screening						
No	1					
Yes	0.89	(0.59‐1.34)	.572			
Initial site of mTNBC						
Lung involvement (vs. no)	2.61	(1.59‐4.29)	**<.001**	2.66	(1.24‐5.70)	**0.012**
Liver involvement (vs. no)	0.75	(0.42‐1.33)	.330			
Bone involvement (vs. no)	0.94	(0.58‐1.54)	.810			
Pleural effusion involvement (vs. no)	0.84	(0.41‐1.72)	.630			
Non‐regional LN involvement (vs. no)	1.61	(0.98‐2.65)	.057			
Hilar/ mediastinal LN involvement (vs. no)	2.41	(1.52‐3.82)	**<.001**	1.70	(1.02‐2.84)	**0.041**
Visceral metastasis						
No	1			1		
Yes	2.10	(1.19‐3.70)	**.010**	0.58	(0.24‐1.42)	0.230
Number of metastatic organ sites						
1	1			1		
≥2	2.94	(1.73‐5.01)	**<.001**	2.28	(1.22‐4.27)	**0.010**
LDH^c^, U/L						
≤250	1					
>250	1.26	(0.78‐2.05)	.340			
CA125^c^, U/mL						
≤35	1					
>35	0.63	(0.37‐1.05)	.076			
CA153^c^, U/mL						
≤25	1					
>25	0.72	(0.42‐1.23)	.230			
CEA^c^, ng/mL						
≤5.20	1					
>5.20	0.74	(0.35‐1.59)	.450			

Abbreviations: BM, Brain metastasis; CA125, Cancer antigen 125; CA153, Cancer antigen 153; CEA, Carcinoembryonic antigenLDH, Lactate dehydrogenase; LN, Lymph node; mTNBC, Metastatic triple negative breast cancer.

^a^ Based on the American Joint Committee on Cancer Staging Manual, 8th ed.

^b^ Patients with primary metastatic disease were not included

^c^ All variables were measured within 2 weeks preceding the first‐line treatment of metastatic disease.

### 3.3 Nomogram construction and validation

**Figure 1:**
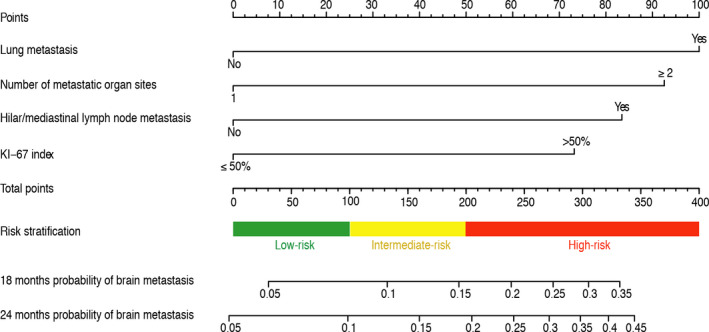
Nomogram predicting subsequent brain metastasis possibility for patients with metastasis triple negative breast cancer

The nomogram (Figure 1) was constructed using the four independent risk factors and scores were assigned for the clinical variables in each subgroup (Table 3). Lung metastasis (no metastasis: score 0; metastasis: score 100) and the number of metastatic organ sites (1 site: score 0; ≥ 2 sites: score 93) contributed the most to the prognosis. Hilar/mediastinal lymph node involvement (no metastasis: score 0; metastasis: score 83) and the KI‐67 index (≤50%: score 0; >50%: score 73) showed a moderate effect on the development of brain metastasis. By summing the scores of each item, we could easily determine the estimated probability of subsequent brain metastasis at each time point.

**Table 3:** Scores of clinical variables in each subgroup

Variables	Points
Lung metastasis	
No	0
Yes	100
Number of metastatic organ sites	
1	0
≥2	93
Hilar/mediastinal lymph node metastasis	
No	0
Yes	83
KI‐67 index	
≤50%	0
>50%	73

**Figure 2:**
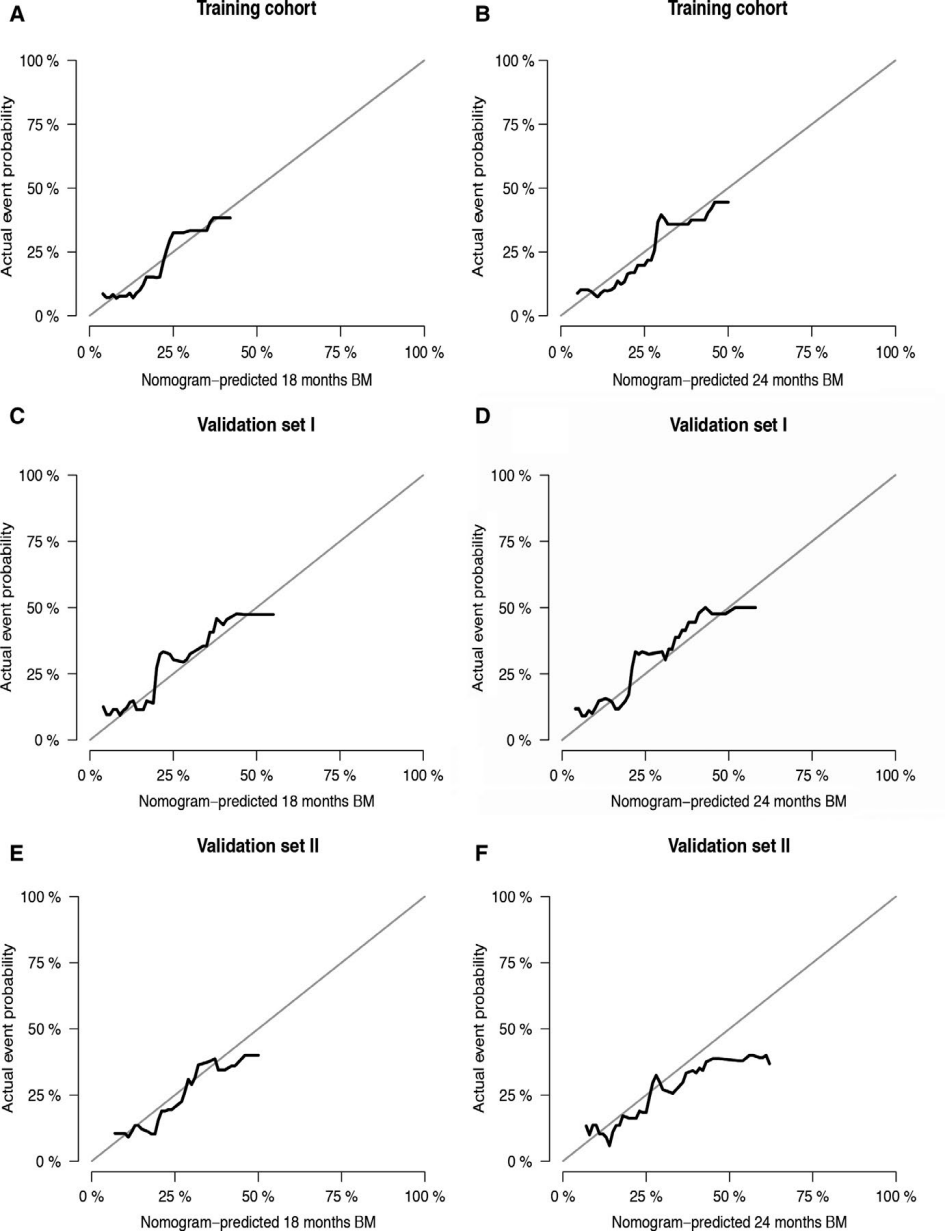
Calibration curves for predicting 18‐months (A) and 24‐months (B) brain metastasis possibility in the training cohort, 18‐months (C) and 24‐months (D) brain metastasis possibility in validation set I and 18‐months (E) and 24‐months (F) brain metastasis possibility in validation set II

The C‐indexes in the training set was 0.74 (0.72 after bootstrap correction) at 18 months and 0.72 (0.70 after bootstrap correction) at 24 months. The C‐index in the validation set I (70.6 [70.4 after bootstrap correction] at 18 months; 71.4 [71.1 after bootstrap correction] at 24 months) and validation set II (68.7 [68.2 after bootstrap correction] at 18 months; 72.4 [72.1 after bootstrap correction] at 24 months) also suggested acceptable predictive accuracy of the model. The calibration plots (Figure 2) demonstrated good consistency between the nomogram‐predicted probabilities and the actual observed rate of brain metastasis at 18 and 24 months among training set, validation set I and validation set II.

### 3.4 Performance of the nomogram in risk stratification

**Figure 3:**
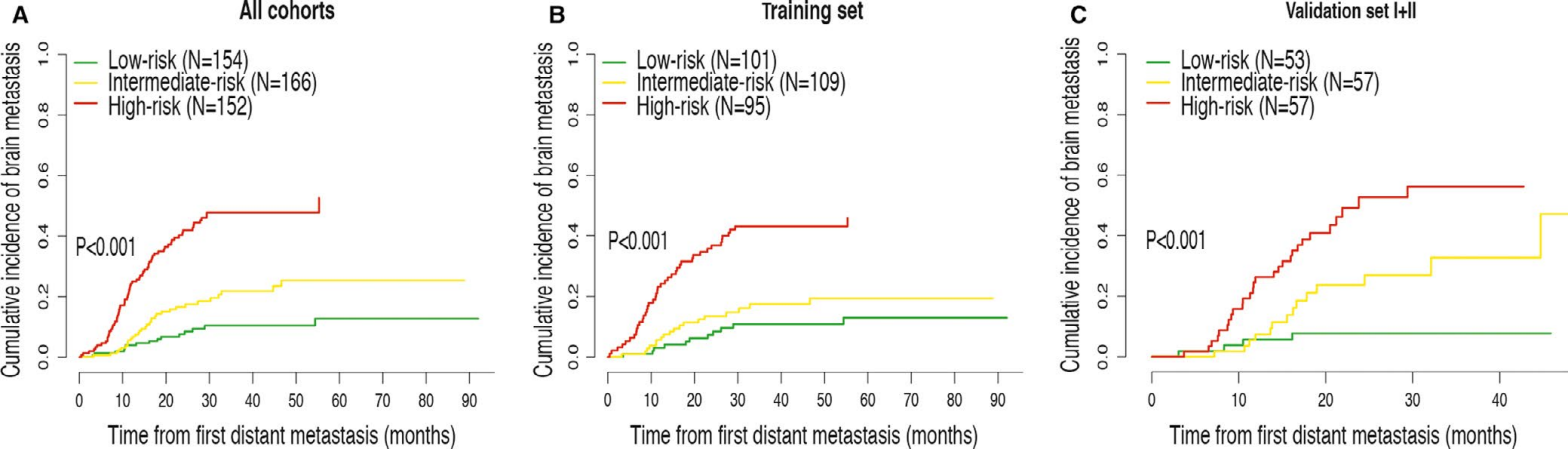
Cumulative brain metastasis incidence curves stratified by risk groups in all cohorts (A), the training cohort (B), and validation set I + II (C)

On the basis of the patients’ total scores from the nomogram, a risk stratification was generated to divide all the patients into three stratums: low‐risk group (154/472, 32.6%; total score < 100), intermediate‐risk group (166/472, 35.2%; 100 ≤ total score < 200), and high‐risk group (152/472, 32.2%; total score ≥ 200) (Figure 1). The cumulative incidence function curves (Figure 3A‐C) indicated that the risk stratification could accurately differentiate the incidence of brain metastasis in the three groups. For all cohorts, the cumulative incidence of brain metastasis at 18 months was 5.3% (95% confidence interval [CI], 2.5%‐9.7%) for patients in the low‐risk group, while 14.3% (95% CI, 9.3%‐20.4%) for patients with intermediate risk and 34.3% (95% CI, 26.8%‐41.9%) for patients with high risk.

### 3.5 Survival benefit of brain MRI screening in stratified risk groups

**Figure 4:**
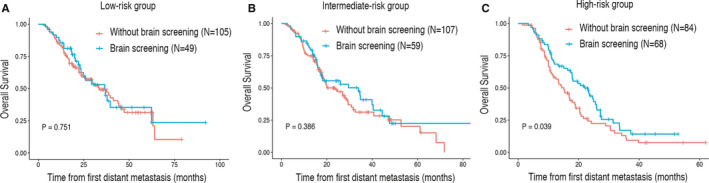
Survival benefit of routine brain MRI screening (every 3‐4 months) in the low‐risk (A), intermediate‐risk (B), and high‐risk (C) groups

To further assess the survival benefit of regular brain MRI screening, Kaplan‐Meier curves were generated in the three risk groups (Figure 4). The results showed that the regular (every 3‐4 months) brain MRI screening could prolong overall survival in high‐risk group (HR 0.67, 95% CI 0.46‐0.98, *P* = .039). However, brain MRI screening did not improve prognosis in the low‐risk group (HR 0.93, 95% CI 0.57‐1.49, *P* = .751) and intermediate‐risk group (HR 0.83, 95% CI 0.55‐1.27, *P* = .386).

## 4 DISCUSSION

In the present study, we included 472 patients and identified four clinicopathological features as prognostic factors. A competing risk nomogram was conducted and validated for predicting brain metastasis in mTNBC patients. C‐index and calibration curves showed that the nomogram had good predictive performance. On the basis of patients’ total points from the nomogram, patients were stratified into three stratums. Survival benefit of brain MRI screening was observed in patients with high risk of brain metastasis. Our study is the first large‐cohort, retrospective study, as far as we know, that developed and validated a competing risk nomogram to predict subsequent brain metastasis in mTNBC patients.

In our findings, clinicopathological features including lung metastasis, number of metastatic organ sites, hilar/mediastinal lymph node metastasis, and KI‐67 index were independent prognostic factors, which were consistent with previous publications. The association between lung metastasis and subsequent brain metastasis was observed in several studies: Slimane et al enrolled patients with MBC and identified lung metastasis as an independent prognostic covariate for brain metastasis in multivariate analysis (hazard ratio [HR] 4.3, 95% CI 1.7‐11.0). Canfeza et al indicated that the presence of lung metastasis at the first metastasis diagnosis was the strongest predictor of subsequent brain metastasis for patients with MBC (odds ratio [OR] 2.82, 95% CI 1.13‐7.00).^24^ Moreover, there is biological evidence that might explain the association between lung metastasis and the subsequent brain metastasis. Stromal cell‐derived factor‐1α (SDF‐1α) is the exclusive ligand for the chemokine ligand‐receptor CXCR4.^25^ CXCR4 is highly expressed in some breast cancer cells,^26^ whereas SDF‐1α is expressed in the brain and lungs,^27^ which causes the breast cancer cells extravasating into the lungs more likely form brain metastasis. The number of metastatic organ sites was also included in the nomogram. Graesslin et al noted that a high tumor burden (>1 metastatic organ sites) was associated with a remarkably higher possibility of subsequent brain metastasis (OR 1.76, 95% CI 1.38‐2.24),^12^ and this association remained valid after analysis of the competing risks.^2^ Hilar/mediastinal lymph node metastasis was also found to be an independent predictor for subsequent brain metastasis in the present study (SHR 1.70, 95% CI 1.02‐2.84). Although it was previously believed that the dissemination of breast cancer cells to the brain occurred through hematogenous metastasis, new evidence showed that a robust lymphatic system might actually exist in brain tissues.^28^ Similarly, the KI‐67 index was also a predictive factor. A retrospective study performed with data from 591 breast cancer patients demonstrated an association between a higher KI‐67 index and a greater risk of subsequent brain metastasis after multivariate adjustment (HR 3.9, 95% CI 1.2‐12.9).^29^ Another study evaluated 198 MBC patients and demonstrated a higher probability of brain metastasis in patients with KI‐67 over‐expression (HR 2.76, 95% CI 1.70‐4.48).^30^

Our prognostic tool can be feasibly used in clinical practice to predict the brain metastasis probability of each individual mTNBC patient. Thus, we hope that it can help clinicians identify patients with high risk of brain metastasis. This may reduce the mortality associated with intracranial disease in several ways. First, identifying the subgroup of patients with a high risk of developing brain metastasis would enrich the populations used in prospective clinical trials (reducing the sample size and costs while maintaining the power) to develop effective preventive interventions for brain metastasis, thus improving the survival outcomes for these patients. Second, risk stratification could also help to implement regular brain MRI screening for patients at high risk, thus, leading to subsequent early detection of asymptomatic brain metastases and early intervention strategies. Our study suggested that brain screening of patients with high risk for brain metastasis might lead to early detection and better survival outcome.

Brain MRI with contrast is routinely recommended for patients with suspicious central nervous system symptoms (unexplained headache, sensory or motoric peripheral or central neurological symptoms). No brain screening is recommended for asymptomatic patients in the National Comprehensive Cancer Network (NCCN) guidelines.^31^ However, compared with symptomatic patients with brain metastasis, asymptomatic brain metastasis patients (diagnosed by screening) showed better survival outcome. A study involving 127 mTNBC patients with brain metastasis showed that the median overall survival was 5.5 months for the symptomatic group and 8.7 months for the asymptomatic group, with a multivariable‐adjusted HR of 1.92 (95% CI 1.13‐3.27).^32^ Moreover, local treatment such as SRS is the first option for the management of brain metastasis.^10^, ^11^ As local treatments are limited to patients with less extensive central nervous system disease, patients might benefit from routine brain MRI screening and early detection of brain metastasis. A prospective study including 200 patients with brain metastasis demonstrated that SRS could achieve local control rates approaching 100% for intracranial metastases less than 6 mm in diameter, thus facilitating earlier detection and prompt treatment.^33^ However, not all mTNBC patients could obtain survival benefit from routine brain MRI screening. It is important to construct a risk stratification tool using all prognostic factors to precisely identify which patient could benefit from screening. Notably, in our prognostic tool, brain surveillance screening could only improve the survival outcome in high‐risk group, but not in low‐ or intermediate‐risk groups. Randomized controlled trials examining the use of brain MRI screening in mTNBC patients are warranted before it can be recommended.

This study has several limitations. The first may be the retrospective nature of our study. Thus, our model requires further prospective studies to verify its accuracy before it can be promoted for widespread use. Second, the triple‐negative subtype was determined based on the primary breast tumor for most of the cases. Therefore, discordance in the molecular subtypes between primary and brain metastatic lesions cannot be ruled out. Third, not all the patients in this study received baseline brain MRI screening. Therefore, patients with asymptomatic brain metastasis at baseline might be wrongly included, which may result in overestimated incidence of subsequent brain metastasis. Fortunately, 72.9% (344/472) patients in our study population received the baseline brain MRI screening. Patients without baseline brain MRI screening made up only 27.1% of the study population. Given the low incidence of brain metastasis as first site of distant relapse, the bias will not have a serious impact on our final results.

## 5 CONCLUSION

We have developed a robust tool that is able to predict subsequent brain metastasis in mTNBC patients. Our model will allow selection of patients at high risk for brain metastasis who might benefit from routine bran MRI screening.

## CONFLICT OF INTEREST

The authors made no disclosures.

## AUTHOR CONTRIBUTIONS

Mingxi Lin: Data curation, formal analysis, investigation, methodology, software, visualization, writing‐original draft, and writing‐review and editing. Yizi Jin: Data curation, formal analysis, investigation, visualization, writing‐original draft, and writing‐review and editing. Jia Jin: Data curation, formal analysis. Biyun Wang: Data curation, formal analysis. Xichun Hu: Project administration, resources, supervision, validation and editing. Jian Zhang: Conceptualization, funding acquisition, formal analysis, methodology, project administration, resources, supervision, validation, writing‐original draft, and writing‐review and editing.

## ETHICAL APPROVAL

All procedures performed in studies involving human participants were in accordance with the ethical standards of the FUSCC Ethics Committee and with the 1964 Helsinki declaration and its later amendments or comparable ethical standards.

## Data Availability

The data will be provided upon the request.
